# The association between subcortical and cortical fMRI and lifetime noise exposure in listeners with normal hearing thresholds

**DOI:** 10.1016/j.neuroimage.2019.116239

**Published:** 2020-01-01

**Authors:** Rebecca S. Dewey, Susan T. Francis, Hannah Guest, Garreth Prendergast, Rebecca E. Millman, Christopher J. Plack, Deborah A. Hall

**Affiliations:** a Sir Peter Mansfield Imaging Centre, School of Physics and Astronomy, University of Nottingham, NG7 2RD, UK; b National Institute for Health Research (NIHR) Nottingham Biomedical Research Centre, Nottingham, NG1 5DU, UK; c Hearing Sciences, Division of Clinical Neuroscience, School of Medicine, University of Nottingham, NG7 2UH, UK; d Manchester Centre for Audiology and Deafness (ManCAD), University of Manchester, Manchester Academic Health Science Centre, M13 9PL, UK; e National Institute for Health Research (NIHR) Manchester Biomedical Research Centre, Central Manchester University Hospitals NHS Foundation Trust, Manchester, M13 9WL, UK; f Department of Psychology, Lancaster University, LA1 4YF, UK; g University of Nottingham Malaysia, Jalan Broga, 43500, Semeniyh, Selangor Darul Ehsan, Malaysia

**Keywords:** Noise induced hearing loss, Functional magnetic resonance imaging, Auditory pathways, Auditory brainstem response, ABR, auditory brainstem response, CN, cochlear nucleus, CSF, cerebrospinal fluid, EEG, electroencephalography, EPI, echo planar imaging, fMRI, functional magnetic resonance imaging, GE, gradient echo, HL, hearing level, IC, inferior colliculus, MGB, medial geniculate body, MNI, Montreal Neurological Institute, MPRAGE, magnetization prepared rapid acquisition gradient echo, MRI, magnetic resonance imaging, SENSE, sensitivity encoding, SPL, sound pressure level, TE, echo time, TR, repetition time, TSE, turbo spin echo

## Abstract

In animal models, exposure to high noise levels can cause permanent damage to hair-cell synapses (cochlear synaptopathy) for high-threshold auditory nerve fibers without affecting sensitivity to quiet sounds. This has been confirmed in several mammalian species, but the hypothesis that lifetime noise exposure affects auditory function in humans with normal audiometric thresholds remains unconfirmed and current evidence from human electrophysiology is contradictory. Here we report the auditory brainstem response (ABR), and both transient (stimulus onset and offset) and sustained functional magnetic resonance imaging (fMRI) responses throughout the human central auditory pathway across lifetime noise exposure. Healthy young individuals aged 25–40 years were recruited into high (n = 32) and low (n = 30) lifetime noise exposure groups, stratified for age, and balanced for audiometric threshold up to 16 kHz fMRI demonstrated robust broadband noise-related activity throughout the auditory pathway (cochlear nucleus, superior olivary complex, nucleus of the lateral lemniscus, inferior colliculus, medial geniculate body and auditory cortex). fMRI responses in the auditory pathway to broadband noise onset were significantly enhanced in the high noise exposure group relative to the low exposure group, differences in sustained fMRI responses did not reach significance, and no significant group differences were found in the click-evoked ABR. Exploratory analyses found no significant relationships between the neural responses and self-reported tinnitus or reduced sound-level tolerance (symptoms associated with synaptopathy). In summary, although a small effect, these fMRI results suggest that lifetime noise exposure may be associated with central hyperactivity in young adults with normal hearing thresholds.

## Highlights

• Neural correlates of lifetime noise exposure using fMRI.
• Auditory brainstem response (ABR) measured in the same listeners.
• Tinnitus and reduced sound-level tolerance measured in the same listeners.
• Enhanced fMRI response to sound onset is associated with high noise exposure.

## 1 Introduction

Noise exposure is the main cause of preventable hearing loss (World Health Organization, 1997). Cochlear damage from noise exposure can lead to increased hearing thresholds, tinnitus (perception of sound with no external source) and diminished sound-level tolerance (Sliwinska-Kowalska and Zaborowski, 2017; Di Stadio et al., 2018). Animals exposed to high sound levels exhibit temporary threshold shifts, which may be accompanied by permanent loss of synapses between inner hair cells and auditory nerve fibers and permanent reduction of wave I of the electrophysiological auditory brainstem response (ABR) (Kujawa and Liberman, 2009). This cochlear synaptopathy may preferentially affect high-threshold auditory nerve fibers (Furman et al., 2013), i.e. fibers thought to encode acoustic information at medium-to-high levels and in background noise (Young and Barta, 1986). Importantly, cochlear synaptopathy can remain “hidden” because the synaptic loss can occur without a permanent hearing threshold shift. Synaptopathy has now been evidenced in mice, rats, guinea pigs, gerbils, chinchillas, and even macaques (Hickox et al., 2017), suggesting a common mechanism in mammals.

It has been hypothesized previously that damage to neural structures precedes hair cell loss, but that this damage may not be revealed by pure tone audiometric thresholds (Zhao and Stephens, 2007). The lack of any diagnostic assessment that is sufficiently sensitive and yet adequately specific has hindered the reliable demonstration of cochlear synaptopathy in humans. Current evidence is mixed. Some studies suggest adults with a history of noise exposure, but with normal hearing as measured by pure-tone audiometry, experience problems with sound discrimination and in particular understanding speech in noise. Noise-exposed workers demonstrated worse speech recognition in multi-talker babble compared to controls (Kumar et al., 2012), and high-noise-risk college students scored lower on word recognition in noise than low-noise-risk counterparts (Liberman et al., 2016). However, other studies found no evidence of a link between noise exposure and speech perception deficits for listeners with normal audiometric thresholds (Grose et al., 2017; Prendergast et al., 2017b; Yeend et al., 2017; Guest et al., 2018a). It may be the case that compensatory behavioral strategies protect performance, especially in high functioning individuals with a normal clinical audiogram, but that nevertheless the effect of synaptopathy in humans might be detected by measurements of physiological function within the central auditory system (Kobel et al., 2017). From animal data, symptoms such as tinnitus and reduced sound-level tolerance in the presence of normal thresholds can potentially be explained by the central gain hypothesis, which states that reduced peripheral auditory input following cochlear damage (for example, synaptopathy) produces a compensatory increase in spontaneous and sound-related activity throughout the ascending auditory pathway (see Auerbach et al., 2014 for a review).

Non-invasive imaging can be used to investigate such pathophysiological mechanisms. ABR waves I-II reflect peripheral auditory function, whilst waves III-V reflect central auditory function. Some studies report associations between ABR wave I amplitude and estimates of noise exposure (Stamper and Johnson, 2015b; Bramhall et al., 2017; Valderrama et al., 2018), whilst others show no discernible relationship between ABR wave I and noise exposure (Fulbright et al., 2017; Grinn et al., 2017; Prendergast et al., 2017a). Some studies have shown that participants with tinnitus have a reduced wave I of the ABR but normal (Schaette and McAlpine, 2011; Gu et al., 2012; Bramhall et al., 2019) wave V. An increased wave V/I ratio is indicative of central gain enhancement. The argument is that reduced peripheral input due to synaptopathy results in enhanced central neural gain, leading to the perception of tinnitus (Schaette and McAlpine, 2011). However, other studies show no association between tinnitus and ABR wave amplitudes (Guest et al., 2017; Shim et al., 2017).

To date, no study has examined the effects of noise exposure using functional magnetic resonance imaging (fMRI). However, physiological correlates of tinnitus and sound-level tolerance have been detected within subcortical structures. Notably, Gu et al. (2010) observed an increased sustained fMRI response in the inferior colliculus (IC) and Medial Geniculate Body (MGB) to continuous broadband noise as a function of decreased sound-level tolerance, which they interpreted as central gain enhancement. It is known that subcortical structures (such as the IC) respond to continuous sounds with a sustained fMRI response, while the response in primary auditory cortex is predominantly transient with phasic peaks immediately after onset and offset (Harms and Melcher, 2002). Therefore, sustained and phasic responses at different positions in the auditory pathway might be differentially sensitive to noise exposure.

This article reports the first investigation of cumulative lifetime noise exposure on ascending auditory pathway function in audiometrically normal adults, as measured by the sustained and transient fMRI response and associated ABR in the same participants. Our primary hypothesis, informed by (Gu et al., 2010) and as pre-registered in Dewey et al. (2018a) was that higher lifetime noise exposure would lead to increased fMRI and ABR responses in central auditory regions compared to lower noise exposure, consistent with central gain enhancement (Gu et al., 2010, 2012; Auerbach et al., 2014) as a consequence of cochlear synaptopathy.

## 2 Materials and methods

A protocol for this study has been published in (Dewey et al., 2018a), as recommended by The Organization for Human Brain Mapping (OHBM) Committee on Best Practice in Data Analysis and Sharing (COBIDAS; Nichols et al., 2017).

### 2.1 Participants

**Fig. 1:**
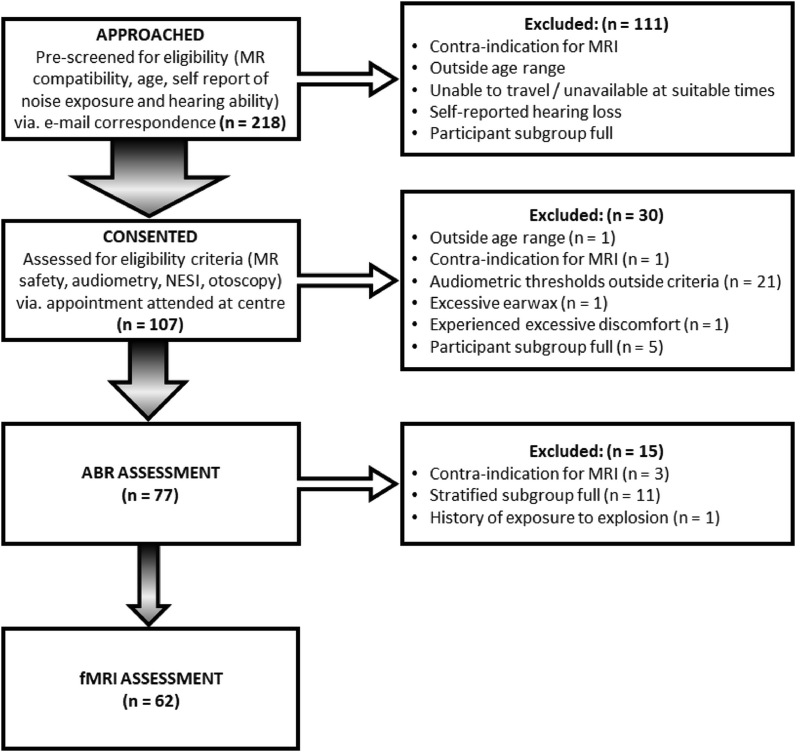
Flow chart showing participant recruitment through the study, detailing the number of participants at each stage and reasons for their exclusion. Contraindications for MRI (n = 3) identified after the eligibility pre-screening stage were due to reasons that were revealed at a subsequent study visit; this included an implant that had previously been thought to be MR compatible, and feelings of claustrophobia prior to the appointment or whilst in the MR scanner.

Experimental procedures conformed to the World Medical Association’s Declaration of Helsinki and were approved by the University of Nottingham School of Medicine Research Ethics Committee (reference: B/1207/2016). Participants aged 25–40 years, and with self-reported normal hearing, were recruited by advertisment across the University, social media and online message boards. A sample size of 60 participants was pre-defined to differentiate fMRI-related activity between noise exposure groups (n = 30 per group), with 80% power (Dewey et al., 2018a). Fig. 1 shows the recruitment of participants through the study and reasons for exclusion. In total, 107 individuals were consented, and 62 met the eligibility criteria for both fMRI and ABR assessments. Key inclusion criteria were normal hearing as defined by hearing thresholds in each ear ≤20 dB HL between 0.5 and 8 kHz and absence of any otological condition as screened by otoscopy and tympanometry. Audiometric thresholds were assessed in a sound-proofed booth using a bespoke calibrated system as described in the protocol (Dewey et al., 2018a). Stimuli were presented using an M-Audio M-Track Quad external sound card (M-Audio, Cumberland, Rhode Island, USA) over Sennheiser HDA300 audiometric headphones suitable for high-frequency audiometry (Sennheiser electronic GmbH & Co. KG, Wedemark, Germany). Stimuli were generated using in-house software written in Matlab (version 2016a, The MathWorks Inc., Natick, Massachusetts). Audiometry was performed using a two-interval, two-alternative forced choice visually cued adaptive paradigm with a two-down one-up rule and a step size of 2 dB. The adaptive procedure was stopped after 12 reversals, and the geometric mean of the signal level at the last eight reversals was computed. This paradigm was used to establish monaural thresholds, in the left ear, followed by the right ear, at frequencies of 0.25, 0.5, 1.0, 2.0, 3.0, 4.0, 6.0, 8.0, 12.0, and 16.0 kHz. Stimuli used at frequencies 250 Hz to 8 kHz were sinusoidal pure tones. Stimuli used at frequencies 12 kHz and 16 kHz were half-octave narrowband noise, to minimize the influence of ear canal resonances and threshold microstructure on measured thresholds. Any participants reporting lifetime noise exposure to heavy weapon firing or explosions were excluded since under these circumstances noise exposure cannot be reliably estimated (Guest et al., 2018c).

Group allocation was based on an estimate of lifetime noise exposure obtained using a beta version of the Noise Exposure Structured Interview (NESI); a comprehensive structured interview which evaluates recreational, occupational/educational, and firearm noise exposure (Guest et al., 2018c). The data collection method in the NESI uses a calendar method which is a widely accepted instrument for enhancing autobiographical recall by providing the respondent with event cues (Glasner and van der Vaart, 2009). In particular, the NESI “provides fields for recording the timing of each exposure period and advises that any contemporaneous life milestones (e.g., graduation or change of workplace) be noted to assist recall” (Guest et al., 2018c, page 4). The NESI has been shown to have sensitivity in the separation of individuals with and without tinnitus, based on noise exposure (Guest et al., 2017), and using robust estimates of noise level (Ferguson et al., 2019), has been shown to reliably provide a coarse estimate of lifetime exposure (Guest et al., 2018b). Further, the variance associated with NESI across participants with a range of lifetime noise exposures is large compared to the error in the estimate of a given individual’s noise exposure (Prendergast et al., 2017b). The cut-off between ‘high’ and ‘low’ noise exposure was pre-specified at 15 units of lifetime noise exposure, equivalent to 85 dB(A) across a full 50-year working lifetime (8 h a day, 5 days a week, 48 weeks a year; National Institute for Occupational Safety and Health, 1998). Noise exposure groups were balanced using age as a stratification variable (25–27, 28–30, 31–33, 34–36 and 37–40 years) (Dewey et al., 2018a), but were chosen to not be balanced for sex since there is no specific hypothesis regarding auditory fMRI responses and sex, thus avoiding issues with an already complex recruitment task (Fig. 1). The Tinnitus and Hearing Survey (THS, Henry, 2015) was used to assess self-reported tinnitus, hearing problems and sound-level tolerance. The presence of tinnitus, hearing problems or reduced sound-level tolerance was defined by a non-zero score (1–4) on any item in the corresponding subscale. Tinnitus intrusiveness was assessed using the intrusiveness subscale of the Tinnitus Functional Index (TFI, Meikle et al., 2012).

### 2.2 Procedure overview

The study consisted of two sessions on separate days. In the first session, participants completed a comprehensive structured interview to estimate lifetime noise exposure and underwent click-evoked ABR testing. In a second session, participants underwent fMRI while listening to a broadband noise stimulus designed to engage cortical and subcortical brain regions throughout the central auditory pathway.

### 2.3 Lifetime noise exposure

The NESI systematically assesses lifetime noise exposure from (1) recreational and (2) occupational and educational noise. For each setting, participants were asked to identify activities they engage in that involve being in an environment estimated to exceed 80 dB(A). The NESI prompts respondents to consider activities experienced across different periods of the lifespan and to use life events as points of reference to improve the quality of recall (Guest et al., 2018c). For each activity, participants were asked to estimate the level of exposure using a vocal effort scale comprising six levels ranging from “raised voice” (87 dB(A)) to “shouting close to listener’s ear” (110 dB(A)) and to estimate the duration for which they were in that environment/engaging in that activity, breaking this down into number of years, number of weeks per year, number of days per week and number of hours per day. For each, participants were asked to recall whether ear protection was used, what type, and the proportion of time for which that ear protection was effective.

Total lifetime noise exposure was calculated for each activity using Equation (1) (Lutman et al., 2008).

(1) $$noise\;exposure=\;\frac{Y\times W\times D\times H}{2080}\times \left[P\times 10^{\frac{L-A-90}{10}}+\left(1-P\right)\times 10^{\frac{L-90}{10}}\right]$$

where *Y* = number of years of exposure, *W* = number of weeks per year of exposure, *D* = number of days per week of exposure, *H* = number of hours per day of exposure, *L* = estimated level of exposure in dB (A), *A* = attenuation of hearing protective equipment (dB), and *P* = proportion of time protective equipment worn (between 0 and 1). Units for all activities were calculated and summed to provide each participant’s total lifetime noise exposure, a measure linearly related to total energy of exposure above 80 dB(A). One unit of noise exposure is equivalent to a working year (8 h a day, 5 days a week, 52 weeks a year = 2080 h) of exposure to 90 dB(A).

### 2.4 fMRI assessment

fMRI was used to assess sound-related responses to broadband noise in brain regions of the ascending auditory pathway comprising the Cochlear Nucleus (CN), Superior Olivary Complex (SOC), Nucleus of the Lateral Lemniscus (NLL), Inferior Colliculus (IC), Medial Geniculate Body (MGB), and auditory cortex.

#### 2.4.1 Stimuli

In-scanner communication, auditory stimulation and ear protection were delivered using an OptoActive Active Noise Cancellation Headphones system (Optoacoustics Ltd., Moshav Mazor, Israel) providing passive attenuation of 24 dB. The fMRI task comprised passive listening to a continuous steady-state broadband noise, filtered using a first-order Butterworth filter between 1.4 and 4.1 kHz, and presented at 85 dB SPL. Following an initial rest period of 64 s, broadband noise was presented for a 24-s ‘on epoch’ followed by 42-s ‘off-epoch’ in a block design. Following an initial 16-s learning period in the first fMRI timeseries, the active noise cancellation reduced the effective scanner sound level to approximately 70 dB SPL (accounting for both passive and active attenuation). This was achieved predominantly by attenuating the fundamental frequencies of the scanner noise, which can be attributed to the readout gradients in the EPI pulse sequence at 1.3 kHz and a mechanical resonance centered around 400 Hz, ensuring that the sound stimulus was clearly audible. During the entire 40-min fMRI study, participants were instructed to attend to a fixation cross presented on a 32” BOLDscreen with a 1920 × 1080 widescreen LCD display (Cambridge Research Systems Ltd., Rochester, UK) positioned behind the scanner and viewed using a mirror attached to the head coil approximately 10 cm from the face.

#### 2.4.2 fMRI data acquisition

fMRI data were acquired on a Philips 3.0 T Ingenia MR scanner (Philips Healthcare, Best, Netherlands) using a 32-element SENSE head coil. Data were collected using a gradient echo (GE) echo-planar imaging (EPI) acquisition at 1.5 mm isotropic spatial resolution, field of view (FOV) of 168 × 168 × 34.5 mm, echo time (TE) of 35 ms; flip angle = 90°; sensitivity encoding (SENSE) factor 2.5; and repetition time (TR) of 2 s. 23 coronal oblique contiguous slices were acquired with equidistant temporal slice spacing and descending slice scan order to provide coverage of the brainstem and Heschl’s gyrus. To optimize placement of the FOV over the ascending auditory pathway, a real-time functional localizer was used to map responses to eight repeats of a 24-s 10-Hz amplitude-modulated broadband noise stimulus followed by 40-s rest periods. This was followed by collection of four 10-min fMRI runs, resulting in a total of 32 cycles (384 ‘sound on’ volumes, and 800 ‘sound off’ volumes) of the broadband noise block paradigm each participant. Breathing and cardiac pulsatility was recorded throughout the fMRI acquisition using respiratory bellows and a peripheral pulse unit attached to the index finger of the left hand (Philips Healthcare, Best, Netherlands) for correction of respiratory and cardiac physiological noise.

Additional EPI volumes were acquired with reversal of the fat-shift direction for image distortion correction, particularly important for alignment of group averaged brainstem fMRI (e.g. Guimaraes et al., 1998). For accurate co-registration of the fMRI EPI data to standard MNI template space, a whole-brain 3D anatomical MPRAGE (Magnetization Prepared Rapid Acquisition Gradient Echo; TE = 2.7 ms, TR = 5.9 ms, flip angle of 8°; and FOV 168 × 168 × 164 mm with reconstructed voxel size 1.5 mm^3^) was acquired with the same spatial resolution and angulation as the GE-EPI fMRI data. In addition, a high-resolution 3D T_2_-weighted Turbo Spin Echo (TSE) anatomical image was acquired (sagittal, TE = 278 ms, TR = 2000 ms, flip angle of 90°; and FOV 249 × 249 × 72 mm with reconstructed voxel size 0.576 mm^3^) on which to overlay the statistical maps.

#### 2.4.3 fMRI data pre-processing

Image pre-processing was performed using FSL software (version 6.0, FMRIB’s Software Library, UK), SPM12 software (Wellcome Trust Centre for Neuroimaging, UK) and in-house software coded in MATLAB. For each individual participant, the fMRI time-series was motion corrected in SPM12. GE-EPI data were then distortion corrected using FSL’s TOPUP algorithm (Andersson et al., 2003; Smith et al., 2004) and corrected for physiological noise using the respiratory and cardiac traces in RETROICOR (Glover et al., 2000). Following this, data were spatially smoothed using a Gaussian kernel of full-width half-maximum 2 mm. Binarized masks of white matter and cerebrospinal fluid were formed from the MPRAGE image using the segmentation tool in SPM12 and threshold at 0.99999. The mean timecourse of white matter and cerebrospinal fluid (CSF) signal within these masks was used as covariates in the general linear model (GLM).

#### 2.4.4 Efficacy of the fMRI preprocessing pipeline

**Fig. 2:**
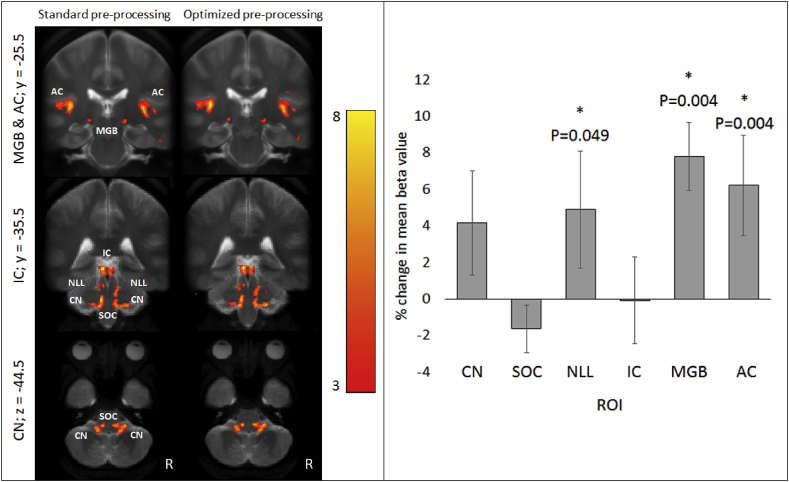
Interim analysis of the influence of distortion and physiological noise correction on sound related activity in the ascending auditory pathway. Left: Group-level (n = 25) sustained sound-related activation for “standard” versus “optimized” pre-processing (p < 0.001 uncorrected, k = 0 voxels) overlaid onto the group-level mean T_2_ turbo-spin echo image. ‘y’ and ‘z’ values denote the MNI slice co-ordinates of the coronal (top) and axial (bottom) images and the color bar denotes T statistic. Right: Group-level mean (±standard error) percent difference in beta values within spherical ROIs calculated for optimized (distortion correction and physiological noise correction) compared to standard pre-processing. A significant increase in beta value (* denotes p ​< ​0.05) is evident in the NLL, MGB and AC ROIs.

As an adjunct to the main research question, we performed a post-hoc interim analysis on a subset of the first 25 participants recruited to the study (9F/16M, aged 31.0 ± 3.9 years) to determine whether the fMRI statistical maps of the sustained fMRI responses (which show greater activity for the auditory brainstem and midbrain structures) were improved by distortion correction and physiological (cardiac and respiratory) noise correction pre-processing steps. Spherical 6-mm ROIs were placed in the CN, SOC, NLL, IC and MGB centered on co-ordinates previously specified by Gutschalk and Steinmann (2015), and the voxel with peak sustained activity in the primary auditory cortex. Within these ROIs, sound-related fMRI responses that were sustained over the 24-s on epoch were examined using a paired *t*-test to determine the combined effect of the pre-processing steps. Random effects analyses were performed on spatially smoothed data analyzed both without (‘standard’ pipeline) and with (‘optimized’ pipeline) distortion and physiological noise correction. Both standard and optimized pre-processing pipelines detected robust sustained group-level fMRI responses throughout the ascending auditory pathway (Fig. 2). The optimized pre-processing yielded a statistically significant improvement (p < 0.05) in the ability to detect group-level sound-related fMRI responses in the NLL, MGB, and AC ROIs, and no detrimental effect in any region (Fig. 2), so these two pre-processing steps were applied to the full study.

Since there have been limited functional studies of subcortical regions, we also evaluated how sample size influences the ability to reliably detect subcortical auditory group responses. To address this, the number of participants used in the sustained response GLM was reduced to 25, 20, 15 and 10, and this result is shown as Supplementary data Table 1S.

#### 2.4.5 fMRI data analysis

fMRI data were analyzed using a random effects GLM (SPM12) computed using successive first- and second-level analyses. The design matrix in the first-level analysis defined the explanatory variables for each individual participant and comprised the (i) transient phasic onset and offset stimulus responses, (ii) sustained stimulus response, (iii) six motion parameters, and (iv) mean white matter and CSF signal time-courses. In this GLM, the phasic responses were encoded as a series of delta functions and the sustained response was encoded as a box-car function, and these were convolved with the hemodynamic response. The phasic and sustained regressors were assessed for orthogonality, and a high degree of orthogonality was found between the onset and offset regressors (−0.08), and onset/offset and sustained regressors (0.11 for both onset/offset). Explanatory variables (iii) and (iv) were considered ‘nuisance’ variables (i.e. potential confounds in the MR signal). The fMRI time-series was high-pass filtered to 1/128 Hz (twice the cycle length) and modeled for temporal autocorrelation across scans with an AR(1) process. Contrast images corresponding to stimulus onset, stimulus offset and the sustained response were generated for each participant. The fMRI response to a continuous stimulus that is perceived as a single event has been shown to vary systematically throughout the auditory pathway from one that is sustained over the stimulus epoch (CN, SOC, NLL, and IC) to one that is phasic with transient peaks at stimulus onset and offset (MGB, auditory cortex) (Gutschalk et al., 2010). This has been interpreted as representing a population neural representation of the beginning and the end of distinct perceptual events that, while weak or absent in the midbrain, begins to emerge in the thalamus and is robust in the auditory cortex. These different auditory response characteristics informed two independent, yet complementary, analyses: i) a second-level voxel-wise analysis of the fMRI contrast images to determine the effect of lifetime noise exposure within individual auditory brain regions, and ii) mixed analysis of covariance (ANCOVA) to determine the effects of lifetime noise exposure across ROIs within the ascending auditory pathway.

Each participant’s MPRAGE image was transformed to the MNI template space in SPM12 (note: the fMRI data was acquired at the same resolution and orientation as the MPRAGE image). This transform computes a matrix for each participant’s MPRAGE image using parameters that best align the template (tissue probability map/atlas) to the individual participant’s image using an affine registration (local optimization) including regularization (penalizing excessive stretching or shrinking) to the MNI symmetric average brain stereotaxic registration model. Following this, the transform was then applied to all contrast images for that participant to move all data into MNI template space. Mean T_2_ TSE maps were then computed by separately co-registering each subject’s T_2_ TSE image to MNI space (the T_2_ TSE images had a different resolution, orientation and FOV to the fMRI data) before averaging across the group.

As described in the protocol (Dewey et al., 2018a), individual contrast images were combined in the second-level GLM of the beta value of the auditory response (representing the magnitude of the stimulus fMRI response) and noise exposure group as a between-subject factor. Voxel-wise statistical significance is reported at p < 0.05 after small volume correction in *a priori* cortical and subcortical ROIs (see Section 2.4.6). In addition, the individual contrast images were interrogated to quantify the average beta value within each ROI on an individual participant basis. To address the primary hypothesis of increased responses in central auditory regions in high lifetime noise exposure compared to low noise exposure, an ANCOVA was performed, with the average beta values in each auditory region and hemisphere as within-subjects factors, noise exposure (low, high) as a between-subjects factor, and de-meaned age as a covariate. Our defined boundary of 15 units of noise exposure, corresponding to the NIOSH distinction between ‘acceptable’ versus ‘at risk’ noise exposure (National Institute for Occupational Safety and Health, 1998), allows for a high vs. low group effect to be studied in noise exposure which itself is a continuous variable. Since the beta values from the two GLMs (onset and sustained) are distinct dependent variables, these measures were used in separate ANCOVAs (note this is a deviation from the protocol paper, in which we stated that responses would be used as levels of a within-subjects factor analysis, which is not a valid statistical analysis).

In an exploratory investigation to examine the association between sound-related activity and noise exposure, a GLM was performed on individual contrast images (both for onset and sustained responses) using noise exposure as a continuous linear regressor, with de-meaned age as a regressor of no interes*t.* Further GLMs were also estimated to address exploratory research questions; these included either tinnitus (present, absent) or reduced sound-level tolerance (present, absent) as the between-subjects factor instead of noise exposure group.

#### 2.4.6 Region of interest (ROI) definition

Use of anatomical landmarks or manual segmentation is challenging for auditory brainstem and midbrain ROIs (Devlin et al., 2006). Instead, a region of interest (ROI) analysis to quantify activity in anatomically defined areas specified in template volume space was performed following the method used by Gutschalk and Steinmann (2015). Subcortical nuclei were determined based on macroscopic anatomy of the average brain, in combination with cross reference to the co-ordinates previously specified by Gutschalk and Steinmann and the contrast images obtained for the ‘sound on versus sound off’ contrast. Auditory cortex was similarly defined using the anatomical boundaries of Heschl’s gyrus/gyri; the superior temporal gyrus and the superior temporal sulcus located lateral and posterior to it, and the ‘sound on versus sound off’ contrast. The ‘sound on versus sound off’ contrast was a summed composite (OR in Boolean algebra) of the three binary images generated by thresholding (p < 0.01 corrected for family-wise error; FWE) the contrast images for stimulus onset, stimulus offset and the sustained responses across all participants (n = 62). Region-specific ROIs for CN, SOC, NLL, IC, MGB and auditory cortex were subsequently created from each sub-region within this binary mask. These ROIs were then used to estimate activity in the subcortical and cortical areas for each noise exposure group from the contrast images estimated in the first-level analysis for each participant.

### 2.5 ABR assessment

The methodology for ABR assessment followed previous work by co-authors (Guest et al., 2017; Prendergast et al., 2017a).

#### 2.5.1 Stimuli

ABR stimuli comprised single-polarity high-pass filtered clicks (using a first-order Butterworth filter with high-pass cut-off of 1.4 kHz) presented at 102 dB peak equivalent SPL. Stimuli were generated using in-house software written in MATLAB (version 2016a, The MathWorks Inc.). Stimuli were presented via shielded Etymotic (Etymotic Research, Inc., Elk Grove Village, Illinois) ER3A transducers with disposable insert foam ear tips. Stimulus presentation was alternated between ears at a rate of 22 Hz (11 Hz per ear) for a total of 7000 clicks per ear.

#### 2.5.2 ABR data acquisition

Electrical activity was recorded using the BioSemi ActiveTwo multichannel electroencephalography (EEG) system with active electrodes (BioSemi BV, Amsterdam, Netherlands). Three channels were used with electrodes attached to the vertex/Cz, right mastoid and left mastoid with 10/20 electrode paste. Two additional electrodes were attached to the forehead (<3 inches apart) to form the ground (Common Mode Sense and Driven Right Leg). Recording was performed in an electrically shielded, darkened, soundproof room, whilst participants lay flat. Participants were instructed to close their eyes, relax, and feel free to fall asleep if able to. Stimuli were presented near-continuously throughout an initial relaxation period prior to recording. Recording commenced when the EEG trace had stabilized, and motion artefacts had subsided. The recording lasted approximately 10 min.

#### 2.5.3 ABR data analysis

ABR data were processed using in-house software coded in MATLAB (Guest et al., 2017; Dewey et al., 2018b). For each participant and for each ear, the time-course of the potential difference between Cz and the ipsilateral mastoid was divided into epochs extending from 10 ms pre-stimulus to 13 ms post-stimulus, after correcting for the 0.91 ms acoustic delay introduced by the tube connecting the transducer to the ear. Epochs with a root-mean-square amplitude of more than 2 standard deviations above the mean were rejected. Data were then averaged across trials, again separately for left and right ear stimulus presentations, and the resulting averaged waveforms were filtered using a fourth-order Butterworth filter between 50 Hz and 1.5 kHz. Filtered averaged waveforms were then baseline-corrected by subtracting the mean amplitude of the 2 ms preceding arrival of the stimulus at the ear drum.

Amplitudes of the peak of ABR waves I and V were quantified to address the primary hypothesis of difference in responses between the low and high noise exposure groups. In addition, the amplitude ratio of waves I/V was computed to provide within-subject normalization and reduce inter-individual variation (Schaette and McAlpine, 2011). Wave I and wave V peaks were identified automatically, using an algorithm that picked out features of the ABR waveform in pre-defined time windows. Peak-picking windows were adjusted slightly from those specified in the protocol, based on observed peak latencies in our cohort (latencies used to develop the protocol were obtained using slightly different methods and equipment). Thus, the peak of wave I was defined as a local maximum falling 1.5–2.5 ms after the calculated arrival time of the stimulus at the ear. If no maximum existed within this window, then the peak of wave I was defined as the highest point within the window. The trough of wave I was defined as the lowest point between 0.3 and 0.8 ms following the wave I peak. The peak of wave V was defined as a local maximum falling between 5.3 and 6.6 ms after the arrival of the stimulus. There were four exceptions (out of 124 ears) where it was necessary to deviate from these rules by altering the time windows in order to successfully characterize one of the peaks: three participants displayed a short wave I, so the relative window for identifying the trough of wave I was between 0.2 and 0.6; one participant exhibited an unusually late wave V so the time window for identification was extended to 7.1 ms after the arrival of the stimulus. To assess any effect of lifetime noise exposure on either ABR wave I or V amplitudes or on wave I/V amplitude ratio, mixed ANCOVA models were specified with noise exposure (low, high) and sex as between-subject factors, and the de-meaned age as a covariate (Van Breukelen and Van Dijk, 2007). Two further ANCOVA models were specified with different between-subjects factors representing (presence/absence of) tinnitus and (presence/absence of) sound-level tolerance.

## 3 Results

### 3.1 Participant characteristics

**Fig. 3:**
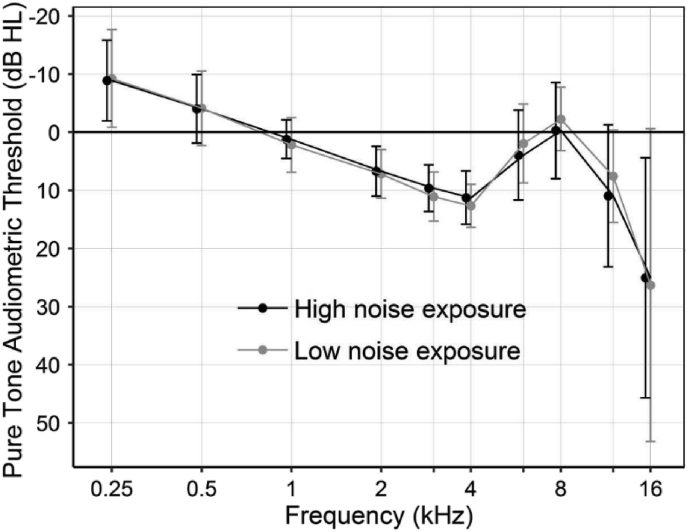
Audiometric threshold (lines denote means and error bars denote standard deviations) over 250 Hz to 16 kHz for low and high exposure groups. Thresholds ≤20 dB HL over the range 500 Hz to 8 kHz were amongst the eligibility criteria for inclusion in the study. 4/60 [low noise exposure group] and 7/64 [high noise exposure group] participants were not measured at 16 kHz as their audiometric thresholds were >90 dB HL (greater than the output level of the audiometer) and as such their 16 kHz values were recorded as 90 dB HL.

Table 1 summarizes the characteristics of the low and high noise exposure groups. All age subgroups comprised at least six participants, with the 28–30 and 31–33 year subgroups each comprising seven participants in the high noise exposure group. Comparison of the baseline characteristics between low and high noise exposure groups found no statistically significant differences in sex (Χ^2^_1_(N = 62) = 3.663, p = 0.056, Table 1) nor audiometric thresholds from 0.25 to 16 kHz (F_1,60_ = 0.100; p = 0.752). These observations at 12 and 16 kHz (F_1,60_ = 0.166; p = 0.685) indicate balanced high-frequency hearing sensitivity (Fig. 3, individual thresholds shown in Fig. 1S of Supplementary data). Audiometric thresholds at 16 kHz could not be measured in those ears in which thresholds exceeded 90 dB HL since the output level of the equipment was limited to this value, and as such were recorded as 90 dB HL for reporting. This accounted for 6 out of 60 ears in the low noise exposure group and 4 out of 64 ears in the high noise exposure group. Although there was an overall trend towards higher thresholds at 4 kHz, in individual participants this dip was too shallow to be defined as a noise-induced (notched) hearing loss (McBride and Williams, 2001). Reports of tinnitus and reduced sound-level tolerance using the THS were more common in the high noise exposure group than low (Χ^2^_1_(N = 62) = 5.963, p = 0.015 and Χ^2^_1_(N = 62) = 7.650, p = 0.006, respectively), with tinnitus perceived as more intrusive in the high noise exposure group (Mann-Whitney U = 359.5, median = 0.0, p = 0.037) (Table 1). However, tinnitus intrusiveness scores were low and would not be interpreted as clinically indicative for either group. Six participants in the high noise exposure group and two in the low noise group experienced both tinnitus and reduced sound level tolerance. Hearing problems as reported in THS responses were equally common across both groups (Χ^2^_1_(N = 62) = 2.517, p = 0.113, Table 1).

**Table 1:** Baseline characteristics of the low and high noise exposure groups. Descriptive statistics of the tinnitus and sound-level tolerance scores are across all individuals including those with a score of 0. Scores on the tinnitus intrusiveness scale range from 0 to 30.

	Low Noise Exposure	High Noise Exposure
Number	30	32
Sex (F/M)	12/18	9/23
Age in years (mean ± st.dev; median; range)	32.0 ± 4.5; 31.0; 25-40	32.0 ± 4.5; 32.5; 25-40
Lifetime noise exposure in units of energy (mean ± st.dev, median, range)	4.0 ± 3.5; 3.6; 0-14	45.0 ± 37.3; 31.0; 15-189
Presence of tinnitus	6	13
Presence of reduced sound-level tolerance	6	10
Presence of hearing problems	13	22
Tinnitus intrusiveness (mean ± st.dev, median, range)	1.2 ± 3.2; 0.0; 0-15	1.9 ± 2.8; 0.0; 0-9

### 3.2 fMRI responses

#### 3.2.1 Robust sound-related responses throughout the subcortical auditory pathway

**Fig. 4:**
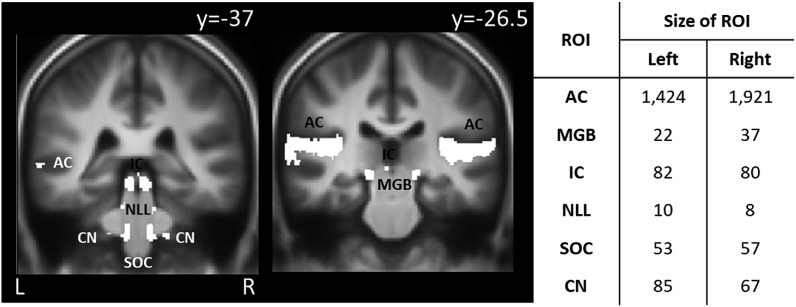
Left: Illustrative coronal slices showing the ascending auditory pathway ROIs as defined from the ‘OR’ combination of binary masks generated from the random effects GLMs of the onset, offset and sustained responses of all (n = 62) participants at p < 0.01 family-wise error (FWE) corrected. ROIs are shown in the cochlear nucleus (CN), superior olivary complex (SOC), nucleus of the lateral lemniscus (NLL), inferior colliculus (IC), medial geniculate body (MGB) and auditory cortex (AC), and overlaid on the group-level mean MPRAGE image (L = left, R = right), ‘y’ denotes the MNI slice co-ordinates. Right: Number of voxels (1.5 mm isotropic) in each ROI by hemisphere.

**Fig. 5:**
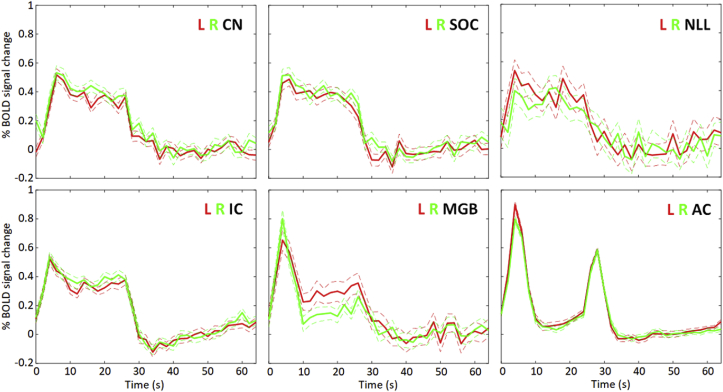
Group mean BOLD percentage change to broadband noise stimulation (all participants, n = 62) in the CN, SOC, NLL, IC, MGB and auditory cortex (AC). Dashed lines show standard error. Note the systematic variation in the fMRI response to the broadband noise stimulus epoch throughout the auditory pathway from one that is sustained over the stimulus epoch (CN, SOC, NLL, and IC) to one that is phasic at stimulus onset and offset (MGB, AC).

**Fig. 6:**
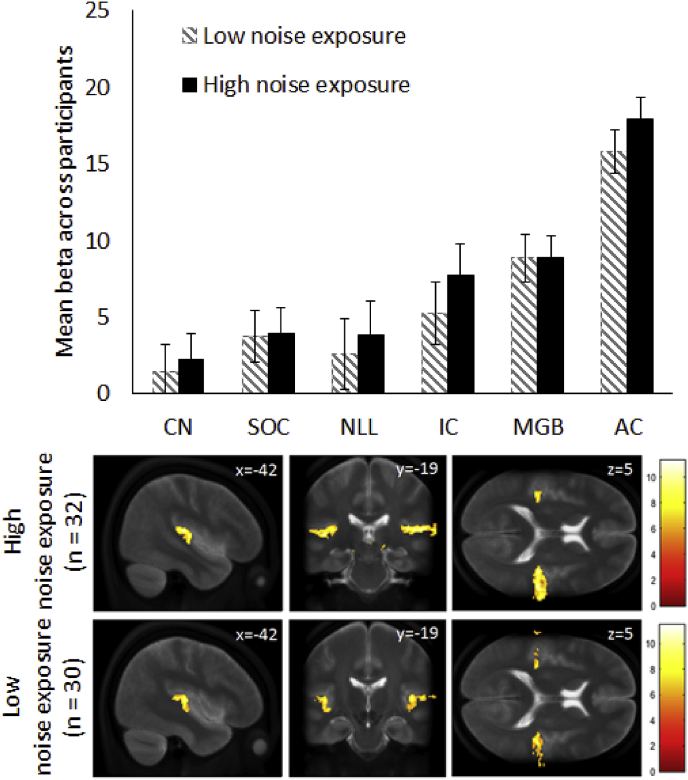
Onset response: estimated marginal mean ROI beta values for stimulus onset in ROIs in low and high noise exposure groups. Beta values represent an average over left and right hemispheres, error bars represent the 95% confidence intervals of the mean. Random effects group activations to the stimulus onset for the low (n = 30) and high (n = 32) noise exposure groups threshold at p < 0.05 FWE corrected with the color bar showing the T statistic. Numbers within the images denote co-ordinates of sagittal, coronal and transverse slices. Statistical maps are overlaid on the mean (n = 62) T_2_ TSE image.

**Fig. 7:**
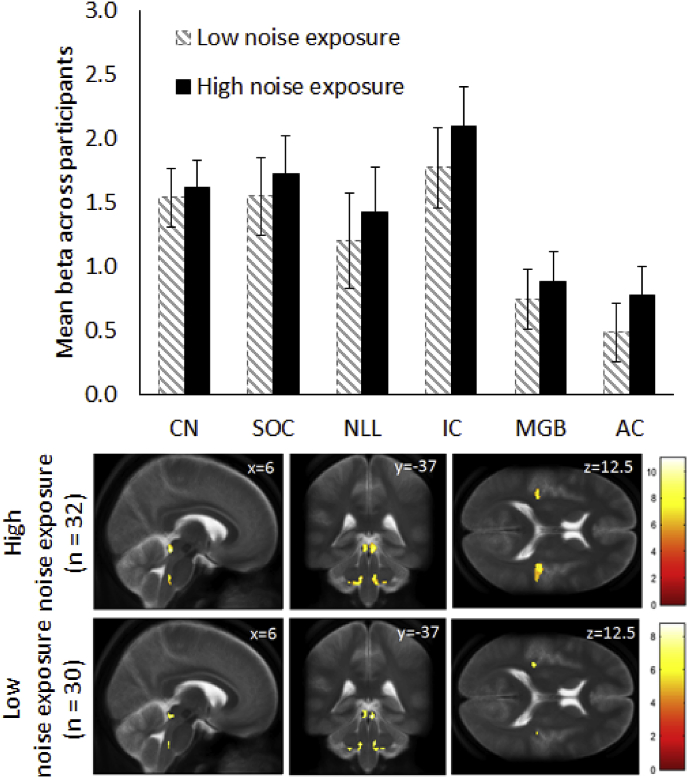
Sustained response: estimated marginal mean ROI beta values for sustained stimulus in ROIs in low and high noise exposure groups. Beta values represent an average over left and right hemispheres, with error bars representing 95% confidence intervals of the mean. Below: random effects group activations to the sustained stimulus for low (n = 30) and high (n = 32) noise exposure groups threshold at p < 0.05 FWE corrected with the color bar showing the T statistic. Numbers within the images denote co-ordinates of sagittal, coronal and transverse slices. Statistical maps are overlaid on the mean (n = 62) T_2_ TSE image.

Group (n = 62) data showed robust activation in response to the broadband noise stimulus. Fig. 4 shows the subcortical and cortical ROIs generated. In agreement with previous reports (Giraud et al., 2000; Harms and Melcher, 2002; and a review article by Nourski and Brugge, 2011), the early ascending auditory pathways (CN and IC) responded predominantly with a sustained response, whilst the auditory cortex showed a strong phasic response to stimulus onset and offset (Fig. 5). Our protocol pre-specified analysis of CN, IC, MGB and auditory cortex, but robust responses were additionally detected in the SOC and NLL, as shown by the ROI time-courses (Fig. 5). Visual inspection shows that the onset of the phasic response is more sensitive to the stimulus features than the offset, particularly for the CN, IC and MGB (and additionally SOC, NLL; Fig. 6, Fig. 7) and that the sustained regressor is a poor match to the shape of the BOLD response in the auditory cortex compared to subcortical regions.

#### 3.2.2 Effect of noise exposure on transient auditory activity in the ascending auditory pathway

Voxel-wise analysis of the contrast images for the transient onset showed greater auditory activity in the high noise exposure group compared to the low noise exposure group, particularly in the right auditory cortex when corrected for multiple comparisons (FWE) at the cluster level (p < 0.05, see Fig. 6). ANCOVA statistics on the ROI analysis showed that lifetime noise exposure is associated with a significant increase in the response to stimulus onset throughout the ascending pathway. An ANCOVA model with noise exposure and region (CN, IC, MGB, auditory cortex) as main factors, and de-meaned age as a covariate, showed that mean beta values were greater in the high than the low noise exposure groups (F_1,59_ = 4.79; p = 0.033) in addition to a significant effect of region (F_3,177_ = 116.99; p < 0.001), but no effect of hemisphere (F_1,59_ = 0.74; p = 0.39). Although the response was greatest in auditory cortex, absence of an interaction between region and noise exposure group (p = 0.39) suggests that the effect of lifetime noise exposure might not be limited to auditory cortex. Including SOC and NLL as two additional regions in the ANCOVA model also gave a significant noise exposure group effect. Note, ANCOVA analysis assumes that all mean beta values are normally distributed, but assessment of kurtosis and skewness in individual ROIs indicated that this was not the case for responses in bilateral CN (p < 0.01) (Field, 2009). All main effects and interactions were confirmed when the CN data were removed, demonstrating that non-normality did not impact the result. An exploratory analysis estimated the GLM using noise exposure as a linear continuous regressor and the transient response as the dependent variable. No brain regions demonstrated a statistically significant linear response. Voxel-wise offset responses were weaker than for the stimulus onset responses (see also Fig. 5), and as such only the onset response was assessed.

#### 3.2.3 Effect of noise exposure on sustained auditory activity in the ascending auditory pathway

Voxel-wise analysis of the contrast images to quantify sustained activity again showed evidence for greater auditory activity in the high noise exposure group than in the low noise exposure group in the right AC when FWE corrected at the cluster level (Fig. 7). An ANCOVA on the sustained response beta values in CN, IC, MGB and auditory cortex ROIs (with all beta values being normally distributed, i.e. exhibiting no significant skew or kurtosis at levels of p < 0.01) showed overall differences in the magnitude of the response across ROIs (F_3,177_ = 59.44; p < 0.001), with the subcortical ROIs, specifically IC, showing the greatest response and auditory cortex the smallest. However, for the sustained response there was a non-significant trend of noise exposure group (F_1,59_ = 3.63; p = 0.06) and hemisphere (F_1,59_ = 2.67; p = 0.11), with no significant interaction between region and noise exposure group (p = 0.65). As above for the transient responses, including SOC and NLL as two additional regions gave the same pattern of results. Again, an exploratory analysis modelling the effect of noise exposure as a linear continuous independent variable did not reveal any significant effects.

#### 3.2.4 Effect of tinnitus and sound-level tolerance on sustained and transient ascending auditory pathway function

Exploratory ANCOVA models with tinnitus or sound-level tolerance as main factors in place of noise exposure group demonstrated no main effect of tinnitus or sound-level tolerance on the sustained response (tinnitus: F_1,59_ = 0.003; p = 0.96; sound-level tolerance: F_1,59_ = 0.25; p = 0.62), or on the onset response (tinnitus: F_1,59_ = 1.19; p = 0.28, sound-level tolerance: F_1,59_ = 0.05; p = 0.83).

### 3.3 ABR results

**Fig. 8:**
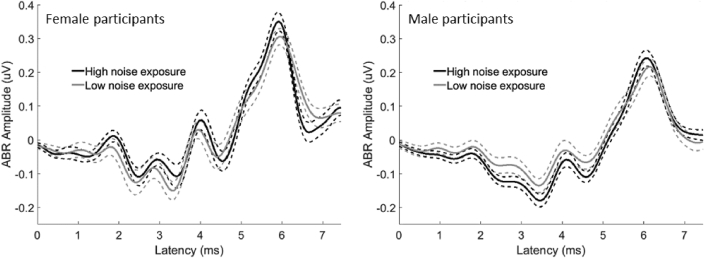
Group-level grand averaged ABR waveforms. Black lines denote the high noise exposure group (n = 32, nine female) and grey lines denote the low noise exposure group (n = 30, 12 female). Solid lines represent the average and dashed lines represent the standard error. In both panels, the grand average was created by first averaging across left and right ears within subjects, and then averaging across subjects.

Visual inspection of the group-level grand averaged waveforms confirmed a typical ABR profile (Fig. 8). There was no significant difference in the amplitudes of wave I and V between the left and right ears across the participant group (ANCOVA F_1,61_ = 0.127; p = 0.723) and no interaction between wave and ear (F_1,61_ = 0.667; p = 0.417). Hence, all subsequent analyses used amplitude estimates averaged across ears.

ABR wave I and V amplitudes followed a normal distribution with no skewness or kurtosis (p > 0.01) (Field, 2009). There was no effect of noise exposure on ABR amplitude (F_1,57_ = 0.456; p = 0.502), nor any effect of tinnitus (F_1,57_ = 2.667; p = 0.108) or sound-level tolerance (F_1,57_ = 1.067; p = 0.306). ABR amplitudes were larger in females than males for both wave I (F_1,57_ = 8.89; p = 0.004) and wave V (F_1,57_ = 14.03; p < 0.001), which may result mainly from sex differences in cochlear mechanical dispersion (Don et al., 1993). There was no interaction between sex and noise exposure group (F_1,57_ = 0.660; p = 0.420). The ratio of wave I/V amplitude was not normally distributed, with both skew and kurtosis (p < 0.001) (Field, 2009). A Mood’s median test was performed as a nonparametric alternative to assess the effect of noise exposure; this revealed no significant difference (p = 0.81; Χ^2^_1_ = 0.06; median = 0.46).

A correlation analysis was run between the magnitude of the fMRI onset response in bilateral NLL (averaged across hemispheres) and wave V of the ABR (averaged across ears), but this was not significant (Pearson’s r = 0.139; p = 0.280; n = 62).

## 4 Discussion

This is the first auditory fMRI evaluation of synaptopathy in humans, here we tested the hypothesis that higher lifetime noise exposure would lead to increased responses in central auditory regions compared to lower noise exposure. fMRI of the ascending auditory pathway was performed in 62 individuals with strictly normal hearing thresholds (≤20 dB HL) from 500 Hz to 8 kHz, allocated to two groups of low and high noise exposure who widely varied in their individual lifetime noise exposures (0–14 vs. 15–189 units). Groups were closely balanced for age (exhibiting the same means, standard deviations and ranges) and high-frequency audiometric thresholds (up to 16 kHz). Although the effect is small, our findings demonstrate for the first time a significant effect of noise exposure on the fMRI response to the onset of a sound stimulus in listeners with apparently normal hearing. Responses throughout the auditory system were greater in individuals with higher lifetime noise exposure levels than in controls with low lifetime noise exposure levels. These enhanced responses to transient stimuli concur with previously published data from animal models of noise exposure (Sheppard et al., 2017, 2018; Schrode et al., 2018). This finding is in agreement with the central gain hypothesis, in which a reduction in neuronal input at the auditory periphery is restored through central compensatory mechanisms (Schaette and McAlpine, 2011; Valderrama et al., 2018), resulting in enhanced cortical responses to an auditory stimulus. The significance of the onset responses has been corrected for multiple comparisons of ROIs, but not against the sustained responses since these are research questions driven by separate hypotheses for their outcomes. These findings now warrant further replication to confirm a more generalized effect.

### 4.1 Comparisons with the published literature in humans

The ABR findings of this study are in agreement with the published ABR literature that does not report an association between noise exposure and ABR waves I or V (Fulbright et al., 2017; Grinn et al., 2017; Prendergast et al., 2017a), but contradicts Stamper and Johnson (2015b) who found an inverse relationship between ABR wave I amplitudes and noise exposure, and Liberman et al. (2016) who found a positive relationship between noise exposure and the ratio between waveform peaks generated by hair cells (the summating potential to action potential ratio, SP/AP). Interestingly, in our fMRI responses we report a positive relation between noise exposure and the physiological fMRI response, which is in-line with Liberman et al. (2016). The disagreement between our ABR and fMRI findings may be due to electrophysiological measures not being sensitive to subclinical noise-induced synaptopathy in humans, and the different origins of the hemodynamic and electrophysiological signals.

The differences between our results and previously published studies may reflect methodological differences. The present study measured audiometric thresholds at extended high frequencies of 12 kHz and 16 kHz, and as such is able to report that these thresholds did not significantly differ between noise exposure groups. In contrast, Stamper and Johnson, 2015a, Stamper and Johnson, 2015b compared audiometric thresholds between noise exposure groups only up to 8 kHz, allowing a potential confound of high-frequency hearing loss between groups. Further, Stamper and Johnson (2015b) used a noise exposure measure that reflected only exposures over the previous year, whereas the present study used a lifetime noise exposure measure. The present study did not have any hypothesis regarding sex of participants and the fMRI response, whereas conversely there is a known relationship between ABR amplitudes and sex, and as such this was a confound in Stamper and Johnson’s original work, which was clarified in a subsequently published letter (Stamper and Johnson, 2015a).

The ABR performed in the present study used a click level of 102 dB peak equivalent SPL. As discussed in Prendergast et al. (2017a), this may not have extensively stimulated all auditory nerve fibers with high characteristic frequencies.

Similarly, some studies investigating associations between electrophysiological ABR measures and tinnitus perception do report a positive association (Schaette and McAlpine, 2011; Gu et al., 2012; Bramhall et al., 2019), whilst others (Guest et al., 2017; Shim et al., 2017) do not. The discrepancy between the present study and the findings of Gu et al. (2012) may be attributed to the exploratory nature of the tinnitus question in the present study and thus the lack of control for confounding factors across groups with and without tinnitus (see Section 4.3).

### 4.2 Considerations of fMRI and ABR findings

The neural coding of stimulus onset is a more dominant feature within the central auditory pathway. Therefore, while central gain might be expected to operate across both onset and sustained responses, there might be greater sensitivity to detect central gain in the transient response. The group difference between low and high noise exposure seen in cortical fMRI responses to stimulus onset (p = 0.033) is of the same order as that observed by Gu et al. (2010) in individuals with reduced sound-level tolerance. This positive fMRI finding counters the often null findings obtained to date using human ABR (Grinn et al., 2017; Guest et al., 2017; Prendergast et al., 2017a), including those reported within this paper. While wave V of the ABR represents activity in the NLL (Ponton et al., 1996), the magnitude of the fMRI onset response in NLL and the amplitude of ABR wave V were not correlated. There are three putative explanations for these results. First, it should be noted that the sample size was powered to detect a change in the fMRI response, rather than ABR. Second, while the ABR directly measures a neuronal response, this is linked to the fMRI signal through a chain of metabolic and hemodynamic processes. As ABR and fMRI measure two distinct physiological phenomena, an effect seen in the hemodynamic response does not necessarily lead to the same pattern in the neuronal response. Third, the data indicated that onset fMRI responses were largely driven by AC activity.

### 4.3 Limitations and future directions

There are several open questions that arise that require further confirmation. While it could be that all significant noise-induced synaptopathy (regardless of susceptibility) is associated with audiometric losses, it is also possible that susceptibility to noise damage is heterogeneous across the population, with some individuals being more susceptible to noise exposure and others more resilient. Susceptible individuals may be those for whom synaptopathy is masked by cochlear damage resulting in audiometric losses, and hence they would not meet eligibility for inclusion in the present study. Such heterogeneity, if present, would certainly reduce our sensitivity for detecting the central effects of noise exposure in participants with clinically normal hearing.

It is currently unknown exactly what factors affect whether noise exposure does or doesn’t lead to synaptopathy in humans, indeed there remains a debate on the origin of hidden hearing loss in humans, and the array of noise types inflicted on human listeners is vast. Consequently, the types of noise exposures reported by participants in the present study varied across individuals. Some participants reported exposure through listening to music (personal stereo, live music events) and others reported exposure to occupational noise from machinery or transport noise, somewhat complicating interpretation of our results. However, this is typical of the field (Xiong et al., 2014; Bramhall et al., 2017; Eggermont, 2017; Kobel et al., 2017; Valderrama et al., 2018). It is possible that the type of noise exposure would affect the impact of noise exposure on fMRI responses, but there is limited information at present about what spectrotemporal features of a sound exposure have the greatest damaging impact on high-threshold auditory nerve fibers. There is relatively recent animal data strongly suggesting that equal energy exposure produces similar synapse loss across different exposure durations (Kujawa, 2019). Therefore, total energy of exposure is thought to be key to inducing a given level of synaptopathy, i.e. the integral over exposure level and duration can be compared directly between exposures of different types, supporting the use of NESI methodology in this study. Impulse noise exposure is known to differently affect auditory nerve fibers, as accounted for in the NESI (Guest et al., 2018c) using kurtosis-correction (Goley et al., 2011), however the NESI does not apply this in a more fine-grained way than differentiating firearm exposure from other exposure types. As such we did not purposively enroll participants according to their dominant type of noise exposure. It is also the case that there is a lack of knowledge about whether noise exposure affects onset or sustained fMRI responses in a linear or non-linear manner, hence our exploratory correlation analysis.

While tinnitus and hyperacusis are both suggested to be associated with increased gain as measured using fMRI from brainstem to cortex (Eggermont, 2017; Eggermont, 2015), our study included too few participants reporting these clinical symptoms to test this hypothesis with statistical rigor (tinnitus n = 19 and reduced sound-level tolerance n = 16), and further study is needed in this area. In addition, our designation into these categories was based on an indicative score obtained from a patient-reported screening test, not a clinical diagnosis. According to the scores obtained using the TFI intrusiveness subscale, even those reporting a score indicative of tinnitus did not appear to be strongly bothered by it and so this subgroup would not constitute clinically significant tinnitus.

The choice of fMRI acquisition was influenced by hardware and software practicalities at the time of the protocol development (Dewey et al., 2018a). We considered both a sparse or clustered-sparse acquisition and continuous acquisition with noise cancellation (Langers et al., 2014; Dewey et al., 2018b), but the continuous acquisition has the advantage of sampling the profile of the hemodynamic response function over the duration of the sound stimulus (Fig. 5), allowing clear definition and separation of stimulus onset and sustained responses. At the time of the study design, the OptoActive Active Noise Cancellation (ANC) system would not apply noise cancellation to a scanning protocol with a sparse or clustered-sparse acquisition. Due to the relatively high spatial resolution (chosen to image the subcortical nuclei) the field of view of the fMRI acquisition was limited to 34.5 mm in the slice direction, precluding any opportunity to observe brain regions outside the temporal lobe, for example the salience network, which may have a significant role in attention during the fMRI task (Damoiseaux et al., 2006). These practical limitations may be overcome in future studies by the implementation of simultaneous multislice acquisitions.

Finally, our study design may have introduced an inadvertent reduction in sensitivity through correlations introduced between the ROI definition method and assessment of the effect of noise exposure through use of the same stimulus condition in both statistical contrasts (Kriegeskorte et al., 2009). However, the ROI locations were entirely independent of the effect of noise exposure and also based on anatomical definitions. Moreover, there were practical comfort limitations which restricted the overall scanning time and this obviated our ability to use a fully independent set of conditions to robustly define the ROIs. We recommend that a future study could use the binary mask devised here for ROI definition (this is provided as Supplementary data).

### 4.4 Optimization of study design, image acquisition and image analysis to improve data quality

We applied Active Noise Cancellation during continuous fMRI acquisition to significantly reduce the impact of acoustic scanner noise. The fMRI protocol acquisition and analysis was optimized to study subcortical auditory responses, with data collected at 1.5 mm isotropic resolution to sample subcortical nuclei, use of a broadband stimulus, and analysis pre-processing steps including distortion correction to improve image quality and normalization of the brainstem at the group level and RETROICOR physiological noise correction to reduce cardiac and respiratory noise (Fig. 2). Previous studies have used cardiac-gated acquisition in combination with sparse fMRI sampling to study subcortical activity, however this considerably limits the spatial coverage and temporal sampling of the data acquisition and consequently statistical power. For example, Gu et al. (2010) were unable to show CN activation at p < 0.01 in the majority of individuals, and Gutschalk and Steinmann (2015) state that “an exact separation of these nuclei is probably beyond the capability of the method”. Several further papers (Smits et al., 2007 Lanting et al., 2008, 2014) report that they were unable to perform fMRI in “subcortical areas, where the motion represents a practical limit in imaging” (Slabu, 2010, pp. 302). Slabu (2010) state that “Because the MGB, CN and SOC were insufficiently activated across subjects, the analysis was focused on the IC and AC”.

Previous fMRI studies have attempted to measure subcortical activity to auditory stimulation. However many studies report group sizes which are likely to be underpowered, thus only able to map activity in some, but not all, of the auditory structures. For example, Slabu (2010) included 10 individuals, while Lanting et al. included 22 (2008) and 29 individuals (2014), and Steinmann and Gutschalk (2012) studied 12 individuals. We show the effect of sample size on the sensitivity to detect group level subcortical responses (see Table 1S, Supplementary data) while recruiting an adequately-powered sample to detect an effect of lifetime noise exposure on the dependent variable. In this study, recruitment was stratified for age in each participant group, with subgroups containing comparable numbers, as outlined prior to commencing the study (Dewey et al., 2018a) and audiometric thresholds were strictly within the clinically normal range and balanced between groups. The latter is often overlooked (Melcher et al., 2000, 2009; Schaette and McAlpine, 2011) and is critical when making comparisons between participant groups (see Guest et al., 2018a for a discussion).

## 5 Conclusions

In summary, this study evaluated ABR and fMRI of the ascending auditory pathway in low and high noise exposure groups. The results suggest that sub-clinical changes resulting from noise exposure in listeners who appear to have ‘normal’ hearing can be detected in humans using non-invasive fMRI optimized for studying the ascending auditory pathways.

## Data and code availability statement

We are willing and able to provide raw and/or processed data and Matlab scripts on request.
