# Supplementary material for: The association between subcortical and cortical fMRI and lifetime noise exposure in listeners with normal hearing thresholds
Source: Neuroimage. 2020 Jan 1;204:116239. doi: 10.1016/j.neuroimage.2019.116239 (PMC6905154; doi:10.1016/j.neuroimage.2019.116239)
Supplement: Multimedia component 2 [file mmc2.docx]

***Supplementary data: Effect of sample size for detection of group level subcortical auditory responses***

Since there have been limited functional studies of subcortical regions, we evaluated how sample size influenced the ability to reliably detect subcortical auditory group responses. To address this additional random effects analyses were performed, with the number of participants used in the sustained response GLM reduced to 25, 20, 15 and 10 by taking a random subsample of the previous group each time (i.e randomly generating 5 numbers between 1 and 25, 20, 15, to determine which participants to remove for subsequent analyses). Table 2 reports cluster- and voxel-level statistics in subcortical ROIs. A sample of 25 participants resulted in statistically significant group activation in each ROI (with the exception of left NLL). With 10 participants, significance was not reached, and with 15 and 20 participants findings were mixed. We conclude that for this broadband noise paradigm, detection of robust activation across the ascending auditory pathway requires a sample size of at least 25 participants. Smaller samples (15-20) may be sufficient if the hypothesis is limited to the IC.

|  | **Number of participants (n)** | | | | | | | |
| --- | --- | --- | --- | --- | --- | --- | --- | --- |
|  | **25** | | **20** | | **15** | | **10** | |
| **Cluster P** | **L** | **R** | **L** | **R** | **L** | **R** | **L** | **R** |
| **MGB** | = 0.001 | = 0.020 | = 0.002 | = 0.250 | n.s. | n.s. | n.s. | n.s. |
| **IC** | < 0.001 | < 0.001 | < 0.001 | < 0.001 | = 0.001 | < 0.001 | n.s. | = 0.001 |
| **NLL** | = 0.119 | = 0.012 | = 0.110 | = 0.060 | = 0.232 | = 0.098 | n.s. | = 0.064 |
| **SOC** | = 0.034 | = 0.001 | = 0.110 | = 0.001 | n.s. | = 0.001 | n.s. | = 0.006 |
| **CN** | = 0.001 | = 0.003 | = 0.002 | = 0.002 | = 0.232 | = 0.005 | n.s. | < 0.001 |
| **Peak T** | | | | | | | | |
| **MGB** | 4.44 | 5.01 | 4.81 | 3.85 | n.s. | n.s. | n.s. | n.s. |
| **IC** | 6.85 | 7.77 | 5.76 | 7.88 | 5.40 | 6.63 | n.s. | 6.60 |
| **NLL** | 4.43 | 6.11 | 5.13 | 4.92 | 4.36 | 3.93 | n.s. | 4.70 |
| **SOC** | 4.81 | 7.11 | 4.20 | 7.76 | n.s. | 8.95 | n.s. | 6.14 |
| **CN** | 5.95 | 5.89 | 5.70 | 6.37 | 4.23 | 5.80 | n.s. | 7.20 |
| **K** | | | | | | | | |
| **MGB** | 11 | 5 | 10 | 2 | n.s. | n.s. | n.s. | n.s. |
| **IC** | 17 | 27 | 15 | 20 | 11 | 13 | n.s. | 8 |
| **NLL** | 2 | 6 | 2 | 3 | 1 | 2 | n.s. | 2 |
| **SOC** | 4 | 13 | 2 | 12 | n.s. | 11 | n.s. | 5 |
| **CN** | 11 | 9 | 9 | 9 | 2 | 7 | n.s. | 14 |

**Table 1S**: The effect of group size (n = 25, 20, 15, 10 participants) on the significance of the sustained responses in subcortical nuclei to broadband noise. Cluster-level significance (Cluster P) and peak voxel T-statistics (Peak T) are shown in spherical ROIs in the ascending auditory pathway. K is the number of supra-threshold voxels reaching a significance of p = 0.001 (uncorrected). Gray background denotes cases where statistically significant bilateral activity is not achieved. n.s. denotes cases where no suprathreshold voxels appeared within the ROI.

**
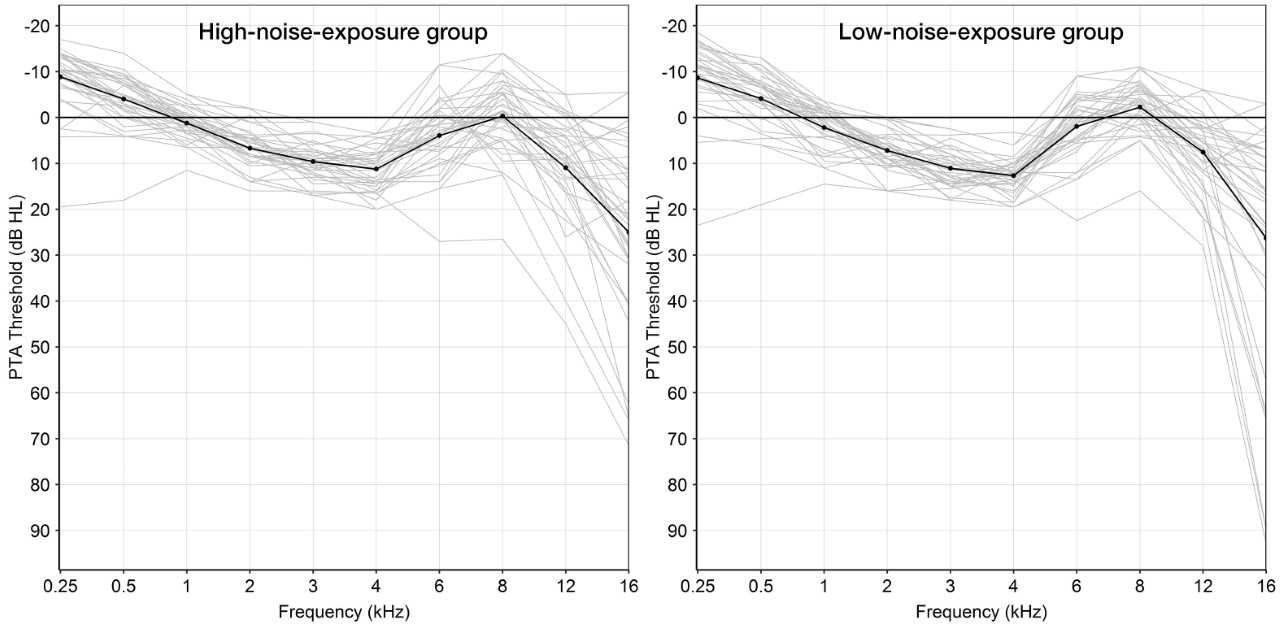
**

**Figure 1S:** Audiometric thresholds of all 62 participants in the study over 250 Hz to 16 kHz for high and low noise exposure groups. Thresholds ≤ 20 dB HL over the range 500 Hz to 8 kHz were amongst the eligibility criteria for inclusion in the study. 4/60 [low noise exposure group] and 7/64 [high noise exposure group] participants were not measured at 16 kHz as their audiometric thresholds were > 90 dB HL (greater than the output level of the audiometer) and as such their 16 kHz values were recorded as 90 dB HL.
